# Editorial: Molecular biomarkers in hepatobiliary and pancreatic cancers: implications of non-coding RNAs and its therapeutic opportunities

**DOI:** 10.3389/fmed.2023.1247320

**Published:** 2023-07-26

**Authors:** Souvick Roy, Milad Moloudizargari, Caiming Xu

**Affiliations:** ^1^Moores Cancer Center, University of California, San Diego, La Jolla, CA, United States; ^2^Beckman Research Institute, City of Hope, Duarte, CA, United States; ^3^Department of General Surgery, First Affiliated Hospital of Dalian Medical University, Dalian, Liaoning, China

**Keywords:** pancreatic cancer, hepatobiliary cancer, molecular biomarker, prognosis, non-coding RNAs

Hepatobiliary and pancreatic cancers, including hepatocellular carcinoma (HCC), gallbladder cancer, cholangiocarcinoma (CCA) and pancreatic ductal adenocarcinoma (PDAC) are lethal malignancies worldwide (1). Despite recent advancements in treatment strategies, the overall survival for patients with hepatobiliary and pancreatic cancers is not essentially improved in past two decades (2, 3). Patients with these malignancies do not present specific symptoms and most of the cases are diagnosed at advanced stages with local or distant metastases, which further contributes to their poor prognosis (4). Besides conventional serological biomarkers (such as CA 19-9, CA 72-4, CEA, AFP etc.) which are routinely used in the clinic (5–7), there is an unmet and urgent need to identify novel molecular biomarkers for diagnosis, prognosis prediction and monitoring response to therapy among patients with hepatobiliary and pancreatic cancers. The Research Topic “*Molecular biomarkers in hepatobiliary and pancreatic cancers: implications of non-coding RNAs and its therapeutic opportunities*” aims to identify potential novel biomarkers at different levels of diagnosis, prognosis and therapy for hepatobiliary and pancreatic cancers.

In this Research Topic, Li C. et al., identified five independent predictors associated with the prognosis of pancreatic cancer patients namely fibrinogen to pre-albumin ratio (FPR), pre-operative controlling nutritional status (CONUT), carbohydrate antigen 19-9 (CA19-9), carcinoembryonic antigen (CEA), and TNM stage and constructed a nomogram using these factors for clinical benefit assessment. It was observed that among all the five independent prognostic factors, FPR had comparable prognostic accuracy as well as significantly associated with overall survival of patients with resectable pancreatic cancer (HR = 2.592, 95% CI: 1.462–4.595, *P* = 0.001). Using decision curve analysis (DCA) and time dependent area under the curve (AUC) analysis, the authors also identified that the nomogram had a better predictive and discriminative ability than the conventional TNM staging system and could potentially serve as a useful tool for prognosis prediction and clinical management of the disease.

In pancreatic cancers, most of the patients are diagnosed at advanced stages with local or distant metastases. Therefore, evaluating prognostic outcome of patients based on the site(s) of metastasis may aid in risk assessment and clinical decision-making. Using the data from the Surveillance, Epidemology and End Results (SEER) database as well as an independent cohort, Zhang et al., showed that PDAC patients with liver metastasis exhibited poor prognosis compared to PDAC patients with lung metastasis. The authors constructed a nomogram model using seven factors including age at diagnosis, location of the tumor, tumor grade, tumor stage, surgical status, presence of metastases in multiple organs as well as treatment with chemotherapy to investigate its ability to predict the prognosis of patients with distant metastases (DM). The authors confirmed that this nomogram can precisely predict the prognosis of PDAC patients with DM in internal and external validation datasets by calculating the ROC curves, C-index, calibration curves and DCA. This will help clinicians to analyze the prognosis of PDAC patients with DM and implement personalized treatment strategies.

In another interesting work, Liu et al., elucidated the characteristics of tumor microenvironment such as infiltration of immune cells, proportion of cancer associated fibroblasts (CAFs) and stemness of tumor cells. The authors performed RNA sequencing from 10 pancreatic cancer patients and identified differentially expressed genes (DEGs) such as BHLHE40, ITGA3, ITGA2, and ADAM9. Furthermore, the authors developed a risk model prediction model based on these four genes and validated their performance to predict the prognosis of pancreatic cancer patients. In addition, the authors identified that the overexpression of BHLHE40 was associated with neoplastic progression by promoting epithelial-mesenchymal transition (EMT) and stemness phenotype. Taken together, this study identified that an increased expression of BHLHE40 is associated with poor prognosis and can be used as a potential therapeutic target in pancreatic cancer. In another study, Chen et al., identified eight necroptosis related genes using publicly available single cell RNA sequencing and transcriptomic datasets and developed a prognostic signature model, namely NCPTS. The authors further classified the patients into high and low risk based on the median risk score value generated based on LASSO regression analysis. It was observed that pancreatic cancer patients with high NCPTS risk score exhibited poor prognosis, low immune infiltration levels and an increased tumor mutational burden (TMB), compared to the NCPTS_low group. Furthermore, using a series of *in vitro* experiments and pancreatic cancer tissue specimens, the authors validated the functional role of one of the key necroptosis related genes, namely ESP8, and identified its role in tumor progression.

The present Research Topic also entails a review article summarizing the interactions between micro RNAs (miRNAs), a type of non-coding RNA and the tumor microenvironment in hepatocellular carcinoma (HCC). The altered expression profiles of miRNAs have been reported to have diagnostic, prognostic, and therapeutic potentials and can be used as molecular biomarkers (8). In this review article by Li J. et al., the authors reviewed the existing literature and summarized the miRNA-associated mechanisms in regulating cell death, mitochondrial function and metabolic reprogramming in HCC. The authors have also explored the clinical usefulness of miRNAs as molecular biomarkers and their prospect for therapeutic applications in HCC.

In summary this Research Topic represents several important and diverse contribution in the field of pancreatic cancer featuring an immune nutritional biomarker, the FPR, the study of prognostic outcomes based on the site of metastasis, BHLHE40 as a molecular biomarker for neoplastic progression and prognosis prediction, and a necroptosis-related gene signature for predicting prognosis. Finally, this Research Topic includes a review article summarizing the regulatory roles of miRNAs in the pathogenesis of HCC and their utility for clinical applications.

## Author contributions

SR drafted the editorial. MM and CX edited and revised the editorial. All authors reviewed and approved the final version.
